# Detection System for U-Shaped Bellows Convolution Pitches Based on a Laser Line Scanner

**DOI:** 10.3390/s20041057

**Published:** 2020-02-15

**Authors:** Yutu Yang, Zengtao Chen, Ying Liu, Yuting Li, Zhongkang Hu, Binli Gou

**Affiliations:** 1 College of Mechanical and Electrical Engineering, Nanjing Forestry University, Nanjing 210037, China; yangyutu@njfu.edu.cn (Y.Y.); lying_new@163.com (Y.L.); liyuting-new@foxmail.com (Y.L.); huzhongkang@163.com (Z.H.); goubinli@163.com (B.G.); 2 Department of Mechanical Engineering, University of Alberta, Edmonton, AB T6G 1H9, Canada

**Keywords:** bellows expansion joint, laser line scanner, S-G algorithm, convolution pitch detection

## Abstract

An expansion joint is mainly composed of bellows and other components; it is attached on a container shell or pipe to compensate for the additional stress caused by temperature differences and mechanical vibrations. In China, the expansion joint fatigue tests are often used to assess the quality of products. After fatigue tests, convolution pitch will be changed. The amount of change is an important index that can be used to evaluate bellows expansion joints. However, the convolution pitch detection is mainly done manually and randomly by inspection agencies before shipping to the end users. This common practice is not efficient and is often subjective. This paper introduced a novel method for automatically detecting the change of the convolution pitch based on a laser line scanner and data processing technology. The laser line scanner is combined with a precision motorized stage to obtain the point cloud data of the bellows. After denoising and fitting, a peak-finding algorithm is applied to search for the crest of a convolution. The method to find the convolution pitch and the decision that needs to be made to ensure product eligibility are described in detail. A DN500 expansion joint is used as a sample to illustrate the efficiency of the system. The application of the technique intuitively allows a higher precision and relative efficiency in quality inspection of bellows expansion joints. It has also been implemented in the Special Equipment Safety Supervision and Inspection Institute of Jiangsu province with great success.

## 1. Introduction

An expansion joint is a connector that can expand under different forms of loading while remaining structurally sound [1,2,3]. It is composed of bellows and other components and can compensate for thermal and mechanical deformations, absorb mechanical vibrations, reduce stress, and increase the average service lives of pipes and tubes. Expansion joints have been widely used in multiple fields, such as petrochemical, steel, aerospace, and nuclear power. The convolution pitch of a bellows expansion joint is an important parameter in quality inspection; it is a key consideration when making the decision of whether bellows expansion joints meet quality requirements. After thousands of tensile and compression fatigue tests, the method of detecting the convolution pitch deformation is usually done manually. It is typically found with a caliper that measures the distance from crest to crest. Then, the collected data is processed to determine the quality of the bellows expansion joint. This type of inspection is overly dependent on manual work, which leads to low efficiency and accuracy. Brazhkin and Mirotvorskii [4] introduced a method for detecting a curved surface using the tangent point of a curve. They used the Coordinate Measuring Machine (CMM) to collect data and extract features from the surface. However, the long measuring cycle and the extremely expensive testing equipment needed for the CMM prevent the quality determination for large-diameter bellows. At the same time, the strict requirements of the detecting environment suggest that this method is not suitable for on-line measurement of bellows expansion joints. 

In order to solve all of the mentioned problems, a system using a laser line scanner (LLS) and a non-destructive measurement method to estimate the quality of expansion joints has recently been built. The system has solved several problems in traditional quality detection methods. Moreover, quality inspection of bellows for special circumstances and requirements (such as bellows expansion joints with nuclear security levels) can significantly advance non-destructive testing techniques. When a laser is projected onto the surface of a bellows, the raw point cloud data is collected automatically via a laser scanner. After noise reduction and curve fitting, the convolution pitch data are found via a peak-searching algorithm. 

The remainder of the paper is organized as follows. The mechanical structure of the laser scanning detection system (LSMS) and the detection procedure are presented in Section 2. In Section 3, the failure judgment algorithm of the test piece is introduced in detail. Section 4 presents the detail of the convolution pitch search method, including point cloud noise reduction, coordinate transformation, curve fitting, and convolution pitch calculation, etc. The application and error analysis are proposed in Section 5. Lastly, the conclusions of our work are given in Section 6.

## 2. LSMS

### 2.1. Design Requirements

According to the standard requirements (obtained from Jiangsu Province Special Equipment Safety Supervision and Inspection Institute) for bellows expansion joints, the developed detecting device needs to use a laser scanner to automatically measure the convolution pitch of the bellows. The data is collected from at least four angles before and after the fatigue test to determine whether the expansion joint is qualified for service or not. Therefore, the bellows expansion joints need to be rotated 360 degrees around the central axis during data collection. The required accuracy of the convolution pitch detection is less than 0.1 mm.

Because the measurement range of the laser scanner is limited, the distance between the scanner and the expansion joint specimen is required to be adjustable. The scanner can move along the radial direction of the expansion joint, and can be adjusted to the proper distance according to the diameter of the bellows.

It is necessary to connect two bellows expansion joints in series for the purpose of fatigue testing. Different specifications of bellows have different lengths. Therefore, the scanner needs to be adjustable in the axial direction of the expansion joint.

### 2.2. Mechanical Structure

**Figure 1:**
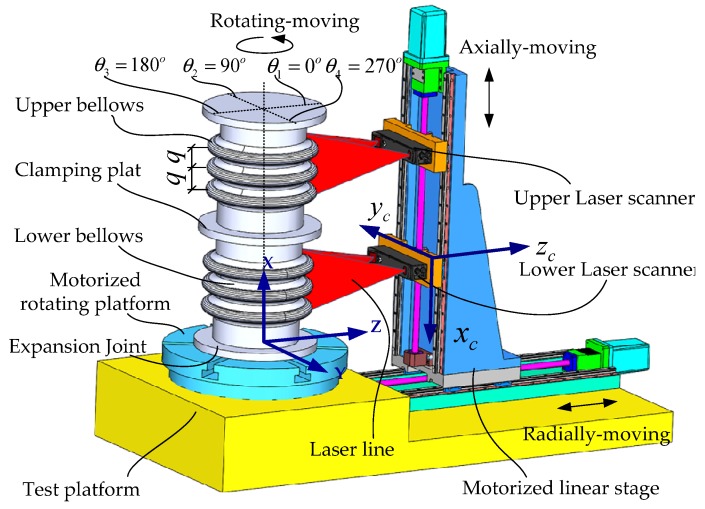
3D model of the laser scanning measurement system.

A 3-D model that lists the detecting device components is shown in Figure 1. The specific mechanical structure of the detecting device includes a radial movement mechanism, an axial movement mechanism, and a rotating movement mechanism. The radial movement mechanism includes a set of ball screws, two sets of rolling linear guides, and a moving platform. The moving platform, which is driven by the ball screws, moved back and forth along the radial direction of expansion joints. The radial movement system mainly guarantees a fixed distance between the laser scanner and the expansion joint. The axial movement mechanism includes a two-way ball screw, two sets of rolling linear guides, two sliders, and two laser scanners (fixed to the slider). The axial movement device drives the laser scanner to move to the appropriate height along the axial direction of the expansion joint. The rotating mechanism includes a motor and a rotating platform which can rotate around its own central axis. The laser scanner can be easily damaged. To avoid damage, a proximity sensor is placed on the laser scanner and a set of limit devices is mounted on the guiding mechanism. In order to save space and for the convenience of the fatigue test, the device adopts a vertical layout. 

In order to achieve online detection, convolution pitch detection and the fatigue test are performed on the same device. This paper does not cover fatigue testing, so the fatigue testing system is not introduced.

### 2.3. Detection Principle

Non-contact methods include optical, electromagnetic, and acoustic measuring devices [5]. Optical measuring devices involve binocular vision, holographic interferometry, and laser triangulation. The laser triangulation method is widely applied for accurate measurement because of its high measurement accuracy, small size device, and fast measurement speed [6,7,8]. So, the laser scanning detection was applied to our system to obtain the outer contour data of the expansion joints of bellows.

As shown in Figure 1, a laser transmitter and laser receiver are mounted onto a laser scanner (model: Gocator 2350) produced by LMI. When a laser beam hits an object, the laser transmitter emits a laser pulse; then, part of the laser energy is returned. The laser energy is then received by the laser receiver. If the returning energy exceeds the preset triggering threshold, the laser scanner calculates its distance from the object. Based on this distance information, the location coordinates of the contours of the object can be calculated.

**Figure 2:**
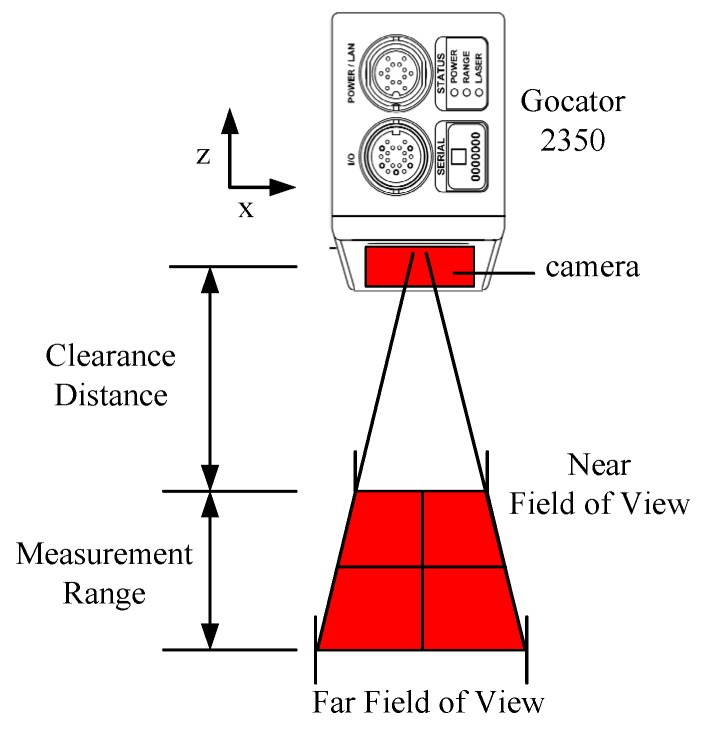
Laser scanner.

As shown in Figure 2, the resolution in the Z-axis direction of the scanner is between 0.019 and 0.060 mm, while in the X-axis direction, it is between 0.150 and 0.300 mm. The total number of single points that the scanner can collect in a single scan is 1280. The field-of-view (FOV) in the X direction is 158 to 365 mm and the measurement range is 400 mm. The clearance distance is 300 mm. 

### 2.4. Detection Procedure

**Figure 3:**
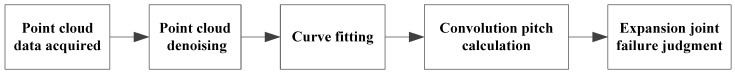
Flowchart of the detection procedure.

First, the outer contour of the bellows expansion joint is automatically scanned before and after the fatigue test. The point cloud data are then transferred to a computer terminal via Ethernet cable. Second, noise reduction is performed on the collected data to eliminate the influence of various noises on the measurement accuracy. The third step is to perform curve fitting on the processed point cloud data to facilitate the search for the peak points. The data of the convolution pitch of the bellows expansion joint is subsequently obtained via a peak-searching algorithm. By comparing the values of the pitch before and after the test, the convolution pitch changes can be seen more intuitively. Then, the condition and performance of the bellows expansion joints can be inferred via the data. Furthermore, the surface of the outer contour is reconstructed by processing the data. Figure 3 is the flowchart of the detection procedure.

## 3. Failure Judgment

**Figure 4:**
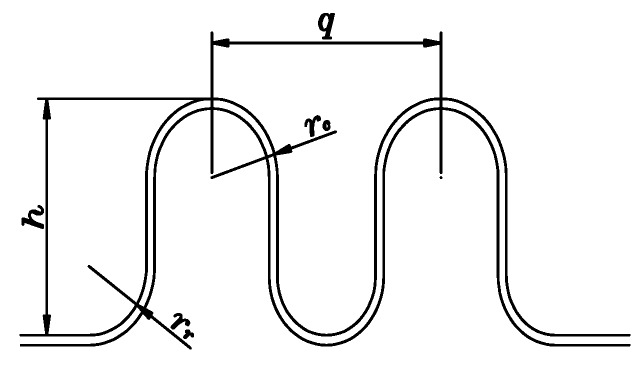
Terminology of the bellows.

As shown in Figure 4, according to the performance standards of bellows expansion joints [9], the main parameters of bellows expansion joints contain the convolution depth h, convolution pitch q, radius $r_{c}$ of the curvature of the crests, and radius $r_{r}$ of the curvature of the troughs [10]. For common bellows, the purpose of the fatigue test is to detect the non-uniformity of the plastic deformation of all of the convolutions after 3000 to 5000 fatigue tests under a certain load. At a certain percentage, the bellows is considered to have failed. The percentage of inhomogeneity that typically requires maximum plastic deformation of the convolution pitch is less than or equal to 15% of the original average convolution pitch. Before the fatigue test, every time that the expansion joints rotate to a certain angle, it triggers the laser scanning of the bellows to obtain the originally measured data, as shown in Table 1. It is assumed here that the bellows has *n* + 1 convolutions and that data are collected from *m* positions. *m* is also the number of scanning instances. As shown in Figure 1, *m* = 4; we define the position of the first scanning angle as $\theta_{1}=0^{{}^{\circ}}$, and then rotate the bellows expansion joint to the second position for the next scanning, where $\theta_{2}=90^{{}^{\circ}}$, and so on. Depending on the value of *m*, the angle of each turn of the bellows expansion joint is also different. Generally, the measurement is performed according to the principle of uniform distribution, that is, the angle of rotation of the bellows expansion joint is the same for each measurement. The total number of convolution pitches is m × n. The calculation of the related parameters is conducted via Equations (1)–(4) [9].

First, the initial value of the average convolution pitch before the fatigue test is determined by

(1) $$\{\begin{array}{c} \bar{q_{s}}=\frac{\sum_{i=1}^{n}\sum_{j=1}^{m}q_{sji}}{m\times n}\\ n\geq 1,m\geq 4 \end{array}$$

where i is the convolution pitch number, j is the position number, n+1 is the total convolution number, m is the position number along the circumference, and $q_{sji}$ is the original value of the convolution pitch, which is equal to the wave distance between the $i^{th}$ convolution and the ${\left(i+1\right)}^{th}$ convolution at the $j^{th}$ position before the fatigue test.

Then, the tension and compression fatigue experiment is performed on the bellows expansion joint. The convolution pitch is measured again by the laser scanner, as shown in Table 2. The value of the deformation and the maximum deformation of a convolution pitch could be determined by Equations (2) and (3), respectively. The maximum circumferential deformation unevenness coefficient of the $i^{th}$ convolution pitch could be determined by Equation (4).

(2) $$W_{ji}=q_{eji}-q_{sji}$$

(3) $$\Delta W_{Ci}=W_{ji\max}-W_{ji\min}$$

(4) $$\tau_{Ci}=\frac{\Delta W_{Ci\max}}{\bar{q_{s}}}\times 100\%$$

where $W_{ji}$ is the value of plastic deformation of the convolution pitch between the $i^{th}$ convolution and the ${\left(i+1\right)}^{th}$ convolution at the $j^{th}$ position after the fatigue test; $q_{eji}$ is the final value of the convolution pitch, which is equal to the wave distance between the $i^{th}$ convolution and the ${\left(i+1\right)}^{th}$ convolution at the $j^{th}$ position after the fatigue test. $W_{ji\max}$ is the maximum value of $W_{1i},\;W_{2i},\;\ldots \;,\;W_{mi}$. $W_{ji\min}$ is the minimum value of $W_{1i},\;W_{2i},\;\ldots \;,\;W_{mi}$. $\Delta W_{Ci}$ is the unevenness value of the $i^{th}$ convolution pitch; $\tau_{Ci}$ is called the maximum circumferential deformation unevenness coefficient (MCDUC) of the $i^{th}$ convolution pitch.

Similarly, the value of the maximum axial deformation of a position could be determined by Equation (5). The maximum axial deformation unevenness coefficient of the $j^{th}$ position can be obtained by Equation (6).

(5) $$\Delta W_{Aj}=W_{ji\max}-W_{ji\min}$$

(6) $$\tau_{Aj}=\frac{\Delta W_{Aj\max}}{\bar{q_{s}}}\times 100\%$$

where $W_{ji\max}$ is the maximum value of $W_{j1},\;W_{j2},\;\ldots \;,\;W_{jn}$. $W_{ji\min}$ is the minimum value of $W_{j1},\;W_{j2},\;\ldots \;,\;W_{jn}$. $\Delta W_{Aj}$ is the axial unevenness value of the $j^{th}$ position; $\tau_{Aj}$ is called the maximum axial deformation unevenness coefficient (MADUC) of $j^{th}$ position.

So, the maximum deformation unevenness coefficient *τ* of a bellows can be obtained by Equation (7). The bellows expansion joints, which fail when *τ* exceeds 15%, are regarded as substandard products.

(7) $$\tau =\max(\tau_{C1},\tau_{C2},\ldots ,\tau_{Cn},\tau_{A1},\tau_{A2},\ldots ,\tau_{Am})$$

## 4. Convolution Pitch Searching

**Figure 5:**
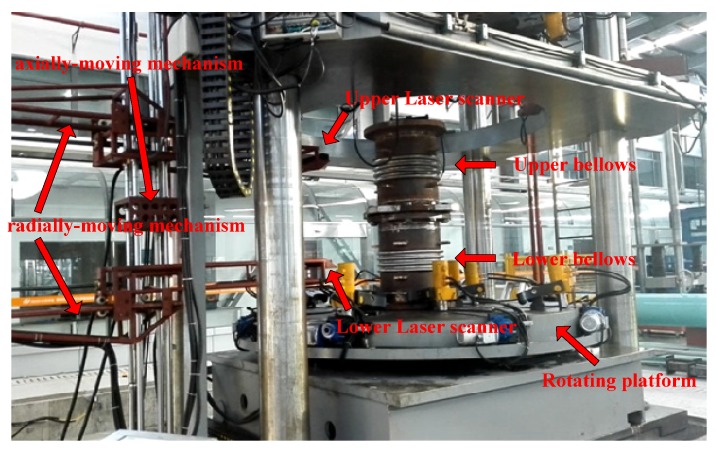
Actual experimental set-up.

An experimental sample containing two bellows expansion joints produced by a pressure vessel manufacturer is shown in Figure 5. The equipment in the photo is an actual experimental device which includes two functions: Convolution pitch detection and fatigue testing. The bellows expansion joint was made of steel B315. The nominal diameter (*DN*) is 500 mm and the total height of the sample is 1500 mm. Each expansion joint has four convolutions and was placed on the rotating platform. When the expansion joint was rotated to a fixed angle, the laser scanner was triggered (by the rotary encoder) to scan the outer contour and obtain convolution pitch data. Then, tension and compression fatigue tests were conducted 3000 times with an internal pressure of 4.0 MPa and with an axial displacement of ±15 mm. After that, to obtain the convolution pitch deformation, the sample was measured by the scanner again.

### 4.1. Denoising

**Figure 6:**
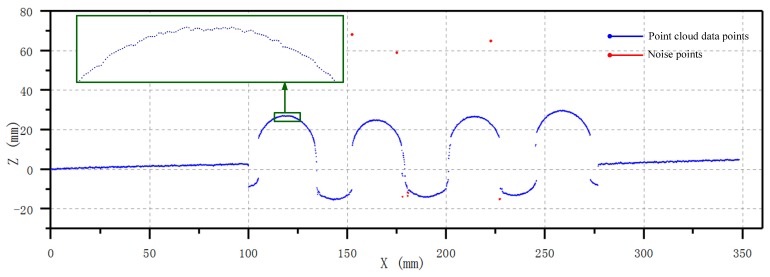
Raw point cloud of the 0° position.

Placing the part on the rotating platform, rotating the platform to a specific angle, and then performing a scan to get 2D point cloud data, the 2D point cloud information of the measurand was obtained. The 2D information of the scanned point cloud data of the 0° position is shown in Figure 6.

The original point data could be read from the LLS using the abovementioned system. The point cloud of each scanning line includes 1280 points. Due to the surface roughness, surface contamination, and material reflection, noise is inevitably produced during detection [11]. Although the output point cloud was preprocessed by the scanning device itself, the noise points contained in each scan line are limited. It can be seen that there was still noise existing in the raw result. Therefore, an effective denoising method is essential for achieving high measurement accuracy. 

The noise points in the original point cloud can be divided into two categories, over-range noise points and outlier noise points, which require different methods for noise reduction. A pass-through filter was used to filter the points that exceeded the measuring range of the sensor affected by the environmental light [12]. Namely, for optical measurement, a pass-through filter served as an efficient method for removing the noise data that were out of range.

The statistical filter could effectively remove the outlier noise points of the 3D point cloud, which were mainly caused by the system noise or surface roughness [12]. However, this filtering method is not suitable for the 2D point cloud. The Savitzky–Golay (S–G) algorithm is one of the effective methods for noise reduction of electronic signals or vibration signals [13,14]. It is a novel method for random noise reduction, in which the S–G filter adopts piecewise weighted polynomials via least-squares estimation. Therefore, effective smoothing is achieved in extracting the original signal from the environmental noise while retaining the shape as closely as possible to the original one. In fact, the 2D point cloud data obtained from each line scanning can be regarded as electronic signals or vibration signals, the X coordinate corresponds to the time axis, the Z coordinate corresponds to the amplitude, the shape of the expansion joint is regarded as the original signal, and the outlier noise points are regarded as noise signals. Then, S–G filters can be used to eliminate the outlier noise points. A large number of subsequent tests have proven that this method has achieved appropriate results in noise reduction.

The S–G filter can be understood as a weighted moving average filter with weight coefficients given as a polynomial of a certain degree. This method requires two key parameters: The window size and the polynomial degree. It is important to choose the window length and the polynomial degrees appropriately to achieve a good compromise between random noise reduction and effective signal preservation [13]. In general, the polynomial degree varies from two to six. When the signal-to-noise ratio (SNR) is high, a low-degree polynomial can be selected; otherwise, a high degree polynomial can be selected. The window length could be adjusted by keeping the polynomial degree as a constant until obtaining an optimal result. Generally, the SNR is usually lower in the part of the contour point cloud data. Therefore, it is assumed in this paper that the polynomial degree is 2, 3, or 4, and the window length is obtained through experiments. 

**Figure 7:**
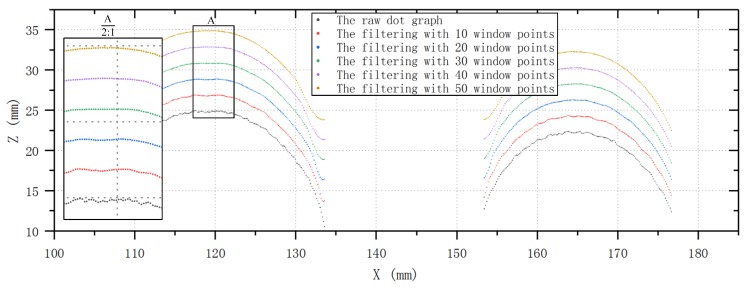
Graph of cubic polynomial S–G filtering with different window points.

**Figure 8:**
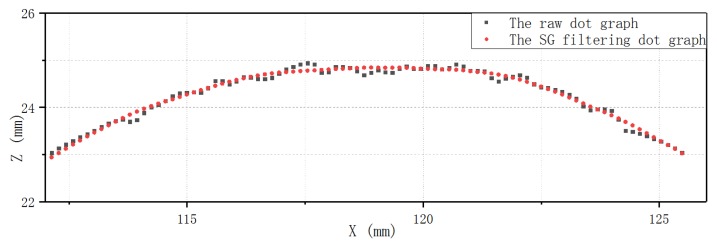
Graph of cubic polynomial S–G filtering with 40 window points and raw dots.

From the discussions of above, the denoising performance of the proposed method was evaluated on the point cloud data of bellows of different diameters. In order to find the appropriate number of window points and polynomial degrees, the window lengths were selected to be 10 points, 20 points, 30 points, 40 points, and 50 points, and the polynomial degrees were 2, 3, or 4, respectively. A large number of denoising tests were performed on the raw point cloud data of bellows of different specifications. Finally, it was found that when the number of window points is 40, a good noise reduction result can be obtained, and the polynomial degree has no obvious effect on denoising. To achieve a comparable compromise between the denoising effect and calculation time, the final selection of window number is 40, and the polynomial degree is 3. Figure 7 show the results of the standard S–G filtering with different numbers of window points, and the polynomial degree is 3. For easy comparison, the Z coordinates of the point cloud data were translated for different window lengths in Figure 7. Figure 8 is a display of two kinds of point graphs of a convolution in the same coordinate system: One is the raw dot graph and the other is a dot graph obtained after filtering using the S–G algorithm. The polynomial degree of the S–G algorithm is cubic and the window width is 40. It can be seen that the proposed method has less signal distortion after denoising and has visibly better smoothing performance. After denoising, the new point of the Z coordinate was obtained by fitting the curve while keeping the X coordinate unchanged, and the point cloud after noise reduction was obtained.

### 4.2. Coordinate Transformation

**Figure 9:**
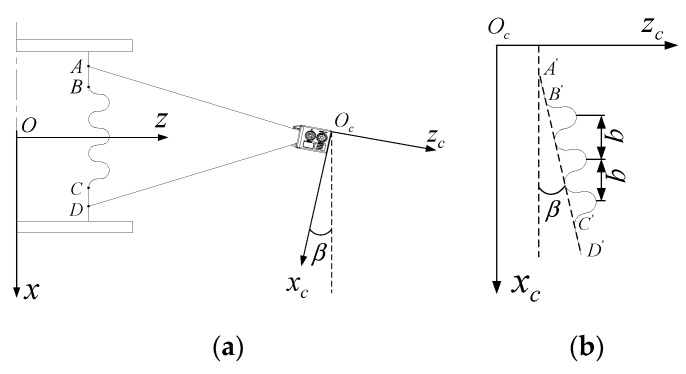
(**a**) Installation error in 3D space; (**b**) point cloud data coordinate relationship.

The installation errors are inevitable in an LSMS, which will affect subsequent convolution pitch detection. The installation errors are mainly generated during mounting of the bellows and the LLS. According to the principle of relative motion, both installation errors can be normalized to the LLS installation errors. The installation errors mainly refer to the fact that the *z_c_*-axis of the laser scanner and the *x*-axis of the bellows is not perpendicular, which will cause the most serious deviations in subsequent convolution pitch calculations. The installation errors are presented in Figure 9. Owing to the fact that the *z_c_*-axis of the laser scanner is not perpendicular to the *x*-axis of the bellows, shown in Figure 9a, the point cloud data obtained by scanning will be deflected, shown in Figure 9b. In order to reduce the deflection error, coordinate transformation should be performed, and the least squares method can be adopted. 

Since the bellows has two cylindrical AB and CD segments at both ends, as shown in Figure 9a, the point cloud data obtained from these two segments can be fitted to a straight line using the least squares method. The fitted straight line equation can be expressed as Equation (8), where *a* is the intercept and *b* is the slope.

(8) $$z_{c}=a+bx_{c}$$

**Figure 10:**
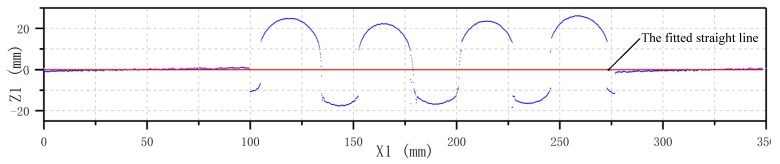
Results of the coordinate transformation of Figure 8.

In order to get the convolution pitch value, the point cloud data need to be coordinate-transformed. The coordinate transformation consists of two basic transformations. The first step is a translation, that is, translating the fitted line along the Z-axis and passing the coordinate origin. The second step is the rotation, that is, rotating the line clockwise to coincide with the X-axis. The new point cloud coordinates after transformation can be obtained by Equation (9). Figure 10 is the result of coordinate transformation using the above method of Figure 6.

(9) $$\left[\begin{array}{c} x\\ z\\ 1 \end{array}\right]=\left[\begin{array}{ccc} 1 & 0 & 0\\ 0 & 0 & 0\\ 0 & -a & 1 \end{array}\right]\left[\begin{array}{ccc} \cos(-\beta ) & \sin(-\beta ) & 0\\ -\sin(-\beta ) & \cos(-\beta ) & 0\\ 0 & 0 & 1 \end{array}\right]\left[\begin{array}{c} x_{c}\\ z_{c}\\ 1 \end{array}\right]$$

where $\left[\begin{array}{ccc} 1 & 0 & 0\\ 0 & 0 & 0\\ 0 & -a & 1 \end{array}\right]$ is a translation matrix, and *a* is the intercept; $\left[\begin{array}{ccc} \cos(-\beta ) & \sin(-\beta ) & 0\\ -\sin(-\beta ) & \cos(-\beta ) & 0\\ 0 & 0 & 1 \end{array}\right]$ is a rotation matrix, and β is the tilt angle of the fitted straight line.

### 4.3. Cubic B-Spline Curve Fitting

After noise reduction and coordinate transformation, in order to find the peak point of convolution, the curve fitting is usually adopted as an effective method. The bellows profile is a free-form curve, and the B-spline is the most effective mathematical representation of the free-form curve. It is widely used in many fields such as computational geometry, image processing, machining, and measuring [15,16,17]. The cubic B-spline curve fitting generates smooth curves with confined errors and acceptable computation times. The experimental results encourage us to generalize this model to the bellows’ profile fitting.

A general *p^th^* degree planar B-spline curve f(t) with *n* + 1 control point $c_{i}=(x_{i},z_{i})\in R^{2}$ can be defined as

(10) $$f(t)=\sum_{i=0}^{n}B_{i,p}(t)c_{i}\;$$

where $B_{i,p}(t)$ is the *i^th^* Bernstein function normalized with the *p^th^* degree defined on the knot vector $U=\{t_{0},t_{1},\ldots ,t_{m+p},t_{m+p+1}\},\;(m+p)\leq n$. In this paper, we investigate the cubic B-spline curve, i.e., *p = 3*; hence, $B_{i,3}(t)$ is the *i^th^* cubic Bernstein basis function.

Given knots $t_{0}<t_{1}<,\ldots ,<t_{m+3}<t_{m+4}$, the cubic Bernstein basis functions are defined as

(11) $$B_{i,0}(t)=\{\begin{array}{c} 1\;if\;t_{i}\leq t<t_{i+1}\\ 0\;otherwise\; \end{array}\;$$

for   p=1,2,3,    i=0,12,3,…,m+3−p

(12) $$B_{i,p}(t)=\frac{t-t_{i}}{t_{i+p}-t_{i}}B_{i,p-1}(t)+\frac{t_{i+p+1}-t}{t_{i+p+1}-t_{i+1}}B_{i+1,p-1}(t)\;$$

It was found that $B_{i,p}(t)$ is only associated with the knots $U=\{t_{i},t_{i+1},\ldots ,t_{i+p+1}\}$. According to the above definition, the curve in $[t_{i},t_{i+1}],\;i=3,\ldots ,m$ is as follows:

(13) $$f_{i}(t)=B_{i,3}(t)c_{i}+B_{i-1,3}(t)c_{i-1}+B_{i-2,3}(t)c_{i-2}+B_{i-3,3}(t)c_{i-3}$$

According to the recurrence formulas (11) and (12), the Bernstein basis function can be represented as follows:

(14a) $$B_{i,3}=\frac{\left(t-t_{i}\right)}{\left(t_{i+3}-t_{i})(t_{i+2}-t_{i})(t_{i+1}-t_{i}\right)}$$

(14b) $$\begin{array}{ll} B_{i-1,3} & =\frac{{\left(t-t_{i-1}\right)}^{2}(t_{i+1}-t)}{\left(t_{i+2}-t_{i-1})(t_{i+1}-t_{i-1})(t_{i+1}-t_{i}\right)}\\ & +\frac{\left(t-t_{i-1})(t-t_{i})(t_{i+2}-t\right)}{\left(t_{i+2}-t_{i-1})(t_{i+2}-t_{i})(t_{i+1}-t_{i}\right)}\\ & +\frac{{\left(t-t_{i}\right)}^{2}(t_{i+3}-t)}{\left(t_{i+3}-t_{i})(t_{i+2}-t_{i})(t_{i+1}-t_{i}\right)} \end{array}$$

(14c) $$\begin{array}{ll} B_{i-2,3} & =\frac{(t-t_{i-2}){\left(t_{i+1}-t\right)}^{2}}{\left(t_{i+1}-t_{i-2})(t_{i+1}-t_{i-1})(t_{i+1}-t_{i}\right)}\\ & +\frac{\left(t-t_{i-1})(t_{i+2}-t)(t_{i+1}-t\right)}{\left(t_{i+2}-t_{i-1})(t_{i+1}-t_{i-1})(t_{i+1}-t_{i}\right)}\\ & +\frac{{\left(t-t_{i+2}\right)}^{2}(t-t_{i})}{\left(t_{i+2}-t_{i-1})(t_{i+2}-t_{i})(t_{i+1}-t_{i}\right)} \end{array}$$

(14d) $$B_{i-3,3}=\frac{{\left(t_{i+1}-t\right)}^{3}}{\left(t_{i+1}-t_{i-2})(t_{i+1}-t_{i-1})(t_{i+1}-t_{i}\right)}$$

Thus, according to the above definition, the Formula (13) can be rewritten as:

(15) $$\begin{array}{ll} f_{i}(t) & =\frac{{\left(t-t_{i}\right)}^{3}}{\left(t_{i+3}-t_{i})(t_{i+2}-t_{i})(t_{i+1}-t_{i}\right)}(c_{i}-c_{i-1})\\ & +\frac{{\left(t-t_{i-1}\right)}^{3}}{\left(t_{i+2}-t_{i-1})(t_{i+1}-t_{i-1})(t_{i+1}-t_{i}\right)}(c_{i-2}-c_{i-1})\\ & +\frac{{\left(t-t_{i+2}\right)}^{3}}{\left(t_{i+2}-t_{i-1})(t_{i+2}-t_{i})(t_{i+1}-t_{i}\right)}(c_{i-2}-c_{i-1})\\ & +\frac{{\left(t-t_{i+1}\right)}^{3}}{\left(t_{i+1}-t_{i-2})(t_{i+1}-t_{i-1})(t_{i+1}-t_{i}\right)}(c_{i-2}-c_{i-3})\\ & +\frac{\left(3t-(t_{i-1}+t_{i}+t_{i+2})\right)}{\left(t_{i+1}-t_{i}\right)}(c_{i-1}+c_{i-2})+c_{i-2}\;(t\in [t_{i},t_{i+1}]) \end{array}$$

Furthermore, the first and second derivatives of $f_{i}(t)$, $t\in [t_{i},t_{i+1}]$ are

(16) $$\begin{array}{ll} f_{i}^{\prime }(t) & =\frac{3{\left(t-t_{i}\right)}^{2}}{\left(t_{i+3}-t_{i})(t_{i+2}-t_{i})(t_{i+1}-t_{i}\right)}(c_{i}-c_{i-1})\\ & +\frac{3{\left(t-t_{i-1}\right)}^{2}}{\left(t_{i+2}-t_{i-1})(t_{i+1}-t_{i-1})(t_{i+1}-t_{i}\right)}(c_{i-2}-c_{i-1})\\ & +\frac{3{\left(t-t_{i+2}\right)}^{2}}{\left(t_{i+2}-t_{i-1})(t_{i+2}-t_{i})(t_{i+1}-t_{i}\right)}(c_{i-2}-c_{i-1})\\ & +\frac{3{\left(t-t_{i+1}\right)}^{2}}{\left(t_{i+1}-t_{i-2})(t_{i+1}-t_{i-1})(t_{i+1}-t_{i}\right)}(c_{i-2}-c_{i-3})\\ & +\frac{3}{\left(t_{i+1}-t_{i}\right)}(c_{i-1}+c_{i-2}) \end{array}$$

(17) $$\begin{array}{ll} f_{i}^{\prime \prime }(t) & =\frac{6(t-t_{i})}{\left(t_{i+3}-t_{i})(t_{i+2}-t_{i})(t_{i+1}-t_{i}\right)}(c_{i}-c_{i-1})\\ & +\frac{6(t-t_{i-1})}{\left(t_{i+2}-t_{i-1})(t_{i+1}-t_{i-1})(t_{i+1}-t_{i}\right)}(c_{i-2}-c_{i-1})\\ & +\frac{6(t-t_{i+2})}{\left(t_{i+2}-t_{i-1})(t_{i+2}-t_{i})(t_{i+1}-t_{i}\right)}(c_{i-2}-c_{i-1})\\ & +\frac{6(t-t_{i+1})}{\left(t_{i+1}-t_{i-2})(t_{i+1}-t_{i-1})(t_{i+1}-t_{i}\right)}(c_{i-2}-c_{i-3}) \end{array}$$

### 4.4. Convolution Pitch Calculation

**Figure 11:**
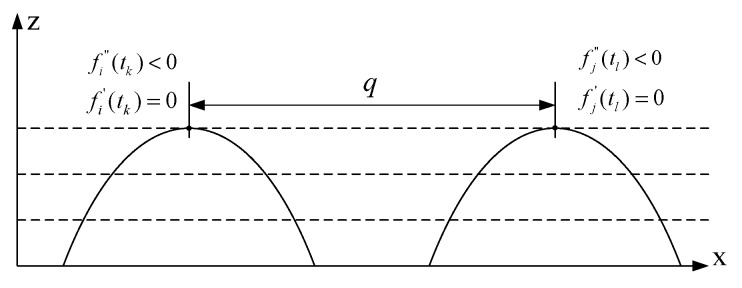
Convolution pitch peak points.

As shown in Figure 11, the convolution pitch *q* is equal to the absolute value of the X-coordinates subtracted from two adjacent convolutions peak points. As long as the X-coordinates of the convolutions peak points can be explored, the convolution pitch *q* can be obtained. There are many peak-searching algorithms with different application fields [18,19,20,21]; e.g., the direct peak-searching method, the Gauss product peak-searching method, and the second-order derivative-searching method. The peak point of the bellows expansion joint fitting curve met the condition that the first derivative was 0 and that the second derivative was negative, that is, $f_{i}^{\prime }(t)=0$ and $f_{i}^{\prime \prime }(t)<0$. Therefore, the second-order derivative searching method is adopted in this paper.

As shown in Figure 11, after two adjacent convolution fitting curves were obtained, the two peak points can be found by using the above derivative-searching algorithm. Then, the convolution pitch *q* is

(18) $$q=\left|t_{k}-t_{l}\right|$$

where $f_{i}^{\prime }(t_{k})=0$, $f_{i}^{\prime \prime }(t_{k})<0$ and $f_{j}^{\prime }(t_{l})=0$, $f_{j}^{\prime \prime }(t_{l})<0$.

## 5. Application and Error Analysis

### 5.1. Application

**Figure 12:**
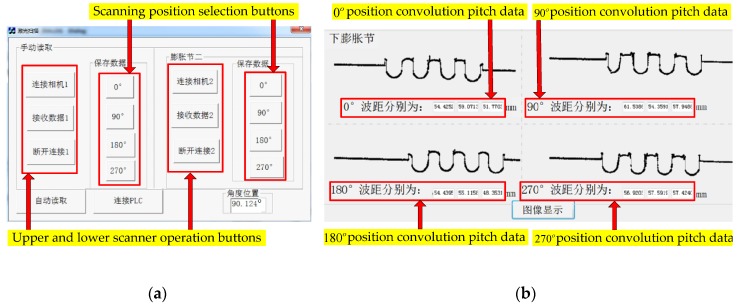
Convolution pitch detection operation and display interface. (**a**) Detection operation interface. (**b**) Raw dot plot and convolution pitch values display interface.

The LSMS mentioned above was evaluated on various bellows with a range of nominal diameter (*DN*) from 500 to 1500 mm. For comparison with manual measurements, a bellows with a nominal diameter of 500 mm was implemented in our experiments. Figure 12 shows the detection operation and the display interface of the convolution pitch values using the LSMS before the fatigue test. The curve in Figure 12b is a raw dot plot, and the convolution pitch values are obtained after noise reduction, curve fitting, and the derivative-searching algorithm. At the same time, in order to compare with manual measurement, we also use a caliper to measure the convolution pitch at the corresponding position before and after the fatigue test of the same bellows expansion joint. The data of the convolution pitch in Table 3 and Table 4 were obtained before and after the fatigue test by the LSMS and the caliper, respectively. 

According to Equation (2), the dimensional change of the convolution pitch before and after the fatigue test of the expansion joint is shown in Table 5. A negative value indicates that the convolution has undergone compressive plastic deformation at this position, while a positive value implies that the convolution has undergone tensile plastic deformation.

According to Equation (1) and the data in Table 3, using the LSMS and calipers to measure the convolution pitch, the calculated $\bar{q_{s}}$ values were 55.83 and 55.73 mm, respectively. According to Equations (4), (6), and (7) and the data in Table 5, we calculated $\tau_{Ci}$ and $\tau_{Ai}$, shown in Table 6 and Table 7. It can be seen from Table 6 and Table 7 that the maximum deformation unevenness coefficients *τ* were 8.82% and 10.1%, respectively. Whether it was measured with a caliper or the LSMS, if the maximum deformation unevenness coefficient did not exceed a maximum limit of 15%, then the expansion joint did not fail, meaning that the expansion joint is a qualified product. That is to say, the result is the same whether measured with calipers or the LSMS.

### 5.2. Comparative Analysis

As shown in Table 5, due to the difference in the accuracy between the calipers and the LSMS, the values of $W_{ji}$ at the same position are almost different from each other. The difference of the $W_{ji}$ using the LSMS and calipers to detect can be expressed by Equation (19).

(19) $$\Delta =\left|L_{W_{ji}}-C_{{}_{W_{ji}}}\right|$$

where $L_{W_{ji}}$ is equal to the value of $W_{ji}$, which is obtained after calculations based on the LSMS detection data; similarly, $C_{{}_{W_{ji}}}$ is equal to the value of $W_{ji}$, which is obtained after calculations based on caliper detection data.

According to Equation (19) and the data in Table 5, the values of Δ at any position are shown in Table 8. The maximum, minimum, and average of Δ are 0.731, 0.013, and 0.37 mm. Compared with the measurement accuracy of calipers, the fluctuation range of Δ is a bit large. We believe that this is mainly due to the lack of a fixed detection datum when using calipers to detect. There is a large random error in the measurement process, that is, traditional detection methods rely heavily on the operator. In addition, $0.37/\bar{q_{s}}=0.37/55.83=0.66\%<<15\%$, the probability of misjudgment using manual measurement is not very high. However, compared with automatic measurement using the LSMS, the efficiency and accuracy of manual measurement are relatively low.

### 5.3. Error Analysis

According to the analysis, as long as the same position data detected before and after the fatigue test are compared, the influence of other measurement errors due to mechanical transmission on the test results can be ignored. So, we did repeated positioning error analyses. This system uses a closed-loop drive system. A 5000-plus rotary encoder was installed under the motorized rotating platform so that the maximum angular error of the bellows would not exceed $0.072^{{}^{\circ}}$, and the nominal diameter range of the detected bellows was from 500 to 1500 mm. The circumferential error range was from 0.628 to 1.884 mm. That is, the deviation of the position detected before and after the fatigue test did not exceed 1.884 mm. Because the shape of the bellows changed continuously, the change of the convolution pitch was very small in the range of arc length of less than 2 mm.

In order to verify the influence of the repeated positioning accuracy of the system on the measurement error, we performed multiple measurements on a bellows expansion joint. Table 9 shows the detection results of a nominal diameter of 500 mm at a position of 270°. To ensure the data is reliable, it must return to the zero degree position after each scan, that is, after each measurement, the bellows is rotated to the 0° position and then rotated to the 270° position for the second measurement. 

Based on the first measurement result, it can be seen from the data in Table 8 that the maximum repeatability error is 0.006 mm. The measurement accuracy required by this detection system is 0.05 mm, so the repeat positioning accuracy of the wave distance measurement system meets the requirements. This result is consistent with the above analysis.

## 6. Conclusions

The primary aim of this paper was to propose a new method for convolution pitch detection of bellows expansion joints. According to the requirements of convolution pitch detection and the external shape characteristics of bellows, a non-contact measuring system based on laser scanning has been developed in the present work. The system was used in the laboratory for more than two years and has been continuously improved. After convolution pitch detection of 34 bellows expansion joints of different specifications, the system tends to be stable and runs well. Compared with traditional manual detection methods, which rely heavily on the operator, several problems have been solved; for example, the detection time is only one third of the manual detection time. At the same time, the proposed method has several other advantages, such as improving the accuracy, realizing online automatic detection, and reducing the labor intensity of detection. However, the convolution pitch cannot be detected dynamically during the fatigue test in the developed LSMS, and there is a certain blind zone in the laser scanning measurement. The binocular vision technology can be employed in the future to eliminate the measurement blind zone and to realize the dynamic detection of bellows expansion joints.

## Figures and Tables

**Table 1:** Data of the convolution pitch of the bellows before the fatigue test.

	Position	θ_1_	θ_2_	θ_3_	θ_4_	…	θ_m_
Convolution Pitch No.							
No.1		*q_s11_*	*q_s21_*	*q_s31_*	*q_s41_*	…	*q_sm1_*
No.2		*q_s12_*	*q_s22_*	*q_s32_*	*q_s42_*	…	*q_sm2_*
…		…	…	…	…	…	…
No.n		*q_s1n_*	*q_s2n_*	*q_s3n_*	*q_s4n_*	…	*q_smn_*

**Table 2:** Data of the convolution pitch of the bellows after the fatigue test.

	Position	θ_1_	θ_2_	θ_3_	θ_4_	…	θ_m_
Convolution Pitch No.							
No.1		*q_e11_*	*q_e21_*	*q_e31_*	*q_e41_*	…	*q_em1_*
No.2		*q_e12_*	*q_e22_*	*q_e32_*	*q_e42_*	…	*q_em2_*
…		*…*	*…*	*…*	*…*	*…*	*…*
No.n		*q_e1n_*	*q_e2n_*	*q_e3n_*	*q_e4n_*	*…*	*q_emn_*

**Table 3:** Data of the convolution pitch before the fatigue test (unit: mm).

	Position	0°		90°		180°		270°	
		LSMS	Caliper	LSMS	Caliper	LSMS	Caliper	LSMS	Caliper
Convolution Pitch No									
No.1		55.425	55.68	61.539	61.28	54.440	54.24	56.920	56.42
No.2		59.071	58.80	54.359	54.42	55.116	55.32	57.592	57.22
No.3		51.770	51.40	57.949	57.82	48.351	48.50	57.424	57.64

**Table 4:** Data of the convolution pitch after the fatigue test (unit: mm).

	Position	0°		90°		180°		270°	
		LSMS	Caliper	LSMS	Caliper	LSMS	Caliper	LSMS	Caliper
Convolution Pitch No									
No.1		55.935	55.86	58.555	58.12	56.380	56.66	56.966	56.88
No.2		56.420	56.88	55.269	55.86	55.843	55.62	58.435	58.24
No.3		52.675	52.36	56.985	56.14	49.978	50.14	58.685	58.46

**Table 5:** Dimensional change of the convolution pitch (unit: mm).

	Position	0°		90°		180°		270°	
Convolution Pitch No		LSMS	Caliper	LSMS	Caliper	LSMS	Caliper	LSMS	Caliper
No.1		0.510	0.18	−2.984	−3.16	1.940	2.42	0.046	0.46
No.2		−2.651	−1.92	0.910	1.44	0.727	0.30	0.843	1.02
No.3		0.905	0.96	−0.964	−1.68	1.627	1.64	1.261	0.82

**Table 6:** Maximum circumferential deformation unevenness coefficient (MCDUC) for different convolutions (unit: %).

	Convolution Pitch No	No.1		No.2		No.3	
MCDUC		LSMS	Caliper	LSMS	Caliper	LSMS	Caliper
$\tau_{Ci}$		8.82	10.1	6.38	6.03	4.64	5.96

**Table 7:** MCDUC for different positions (unit: %).

	Position	0°		90°		180°		270°	
MADUC		LSMS	Caliper	LSMS	Caliper	LSMS	Caliper	LSMS	Caliper
$\tau_{Ai}$		6.37	5.17	6.97	8.25	2.17	3.80	2.18	1.00

**Table 8:** Data of Δ at different positions (unit: mm).

	Position	0°	90°	180°	270°
Convolution Pitch No		Δ			
No.1		0.33	0.176	0.48	0.414
No.2		0.731	0.53	0.427	0.177
No.3		0.055	0.716	0.013	0.441

**Table 9:** Convolution pitch data of multiple measurements at the 270° position (unit: mm).

	Measurement No	1	2	3	4	5	6	7	8
Convolution Pitch No									
No.1		56.920	55.924	55.926	55.922	55.915	55.920	55.922	55.923
No.2		57.592	57.596	57.595	57.588	57.590	57.591	57.593	57.594
No.3		57.424	57.428	57.422	57.420	57.425	57.420	57.430	57.428
